# Comparative Study of the Leaf Volatiles of *Arctostaphylos uva-ursi* (L.) Spreng. and *Vaccinium vitis-idaea* L. (Ericaceae)

**DOI:** 10.3390/molecules15096168

**Published:** 2010-09-02

**Authors:** Niko Radulović, Polina Blagojević, Radosav Palić

**Affiliations:** Faculty of Science and Mathematics, University of Niš, 18000 Niš, Serbia; E-Mails: blagojevicpolina@gmail.com (P.B.); radosavpalic@yahoo.com (R.P.)

**Keywords:** *Arctostaphylos uva-ursi* (L.) Spreng., *Vaccinium vitis-idaea* L., essential oil, α-terpineol, linalool, chemometrics

## Abstract

The first GC and GC/MS analyses of the essential oils hydrodistilled from dry leaves of *Arctostaphylos uva-ursi* and *Vaccinium vitis-idaea* enabled the identification of 338 components in total (90.4 and 91.7% of the total GC peak areas, respectively). Terpenoids, fatty acids, fatty acid- and carotenoid derived compounds were predominant in the two samples. Both oils were characterized by high relative percentages of α-terpineol and linalool (4.7-17.0%). Compositional data on the volatiles of the presently analyzed and some other Ericaceae taxa (literature data) were mutually compared by means of multivariate statistical analyses (agglomerative hierarchical cluster analysis and principal component analysis). This was done in order to determine, based on the essential oil profiles, possible mutual relationships of the taxa within the family, especially that of species from the genera *Arctostaphylos* and *Vaccinium*. Results of the chemical and statistical analyses pointed to a strong relation between the genera *Vaccinium* and *Arctostaphylos*.

## 1. Introduction

It has been known for centuries that leaves of *Arctostaphylos uva-ursi* (L.) Spreng., Ericaceae (Bear's grape, bearberry) possess powerful astringent activity, mainly due to the presence of glycosides such as arbutin [1]. In 1601, Clusius reported its earlier use by Galen (*ca.* 130–200 C.E.) as a hemostatic. In modern Western medical practice, its use seems to begin with Spanish and Italian physicians (*ca.* 1730–1740 C.E.) for calculus complaints [2]. For more than 100 years now this plant species has been official in nearly all Pharmacopeias and is widely used for treating bladder and kidney disorders, inflammatory diseases of the urinary tract, urethritis, cystitis, for strengthening and imparting tone to the urinary passages, *etc.* [1,2]. During collection, bearberry is commonly confused with cowberry (*Vaccinium vitis-idaea* L.) and box (*Buxus sempervirens* L.). Poisonous *B. sempervirens* (similar morphological characteristics, but without the pharmacologically active arbutin) has occasionally been used to adulterate the drug [1,3]. *Vaccinium vitis-idaea* and other representatives of the genus *Vaccinium* (Ericaceae), on the other hand, could be regarded as suitable substitutions for *A. uva-ursi* (comparable content of arbutin and similar pharmacological action of leaves’ infusions) [1]. Some recent studies have shown that biologically active phenologlycosides, simple phenols, flavonoids, tannins, polysaccharides, etc. are also present in *V. vitis-idaea* and *A. uva-ursi* [4,5,6,7,8].

It has been previously shown that essential oils may, despite their small yield, contribute to the medicinal properties of the plant [9]. Moreover, volatile metabolites could be potentially used as a tool which could give a quick insight to the presence/absence (*i.e.* expression) of a certain biosynthetic “apparatus” in some plant taxa, and to some extent, to the (dis)similarity of the compared species on a molecular level [10]. To the best of our knowledge, there are no previous reports on the essential oil profile of *A. uva-ursi* and there is a limited data concerning the volatiles of *V. vitis-idaea* and only of the berries [11,12]. Thus, the aim of this work was set to analyze in detail (using GC and GC/MS) and compare the chemical composition of the essential oils hydrodistilled from the dry leaves of *A. uva-ursi* and *V. vitis-idaea*, in order to determine if any further phytochemical similarities between the two species exist. Comparison of the compositional data of the oils from a number of Ericaceae taxa (present study and the literature data [13,14,15,16,17,18,19,20,21,22,23]) was achieved using multivariate statistical analyses (MVA: agglomerative hierarchical cluster analysis (AHC) and principal component analysis (PCA)).

## 2. Results and Discussion

GC and GC/MS analyses of the essential oils extracted from *Arctostaphylos uva-ursi* and *Vaccinium vitis-idaea* leaves enabled the identification of 338 different constituents (243 in *A. uva-ursi* and 187 in *V. vitis-idaea*, Table 1), representing 90.4 and 91.7% of the total GC peak areas, respectively. The major contributors to the *V. vitis-idaea* oil were α-terpineol (17.0%), pentacosane (6.4%), (*E*,*E*)-α-farnesene (4.9%), linalool (4.7%) and (*Z*)-hex-3-en-1-ol (4.4%). The same two constituents, α-terpineol (7.8%) and linalool (7.3%), were predominant in the oil of *A. uva-ursi*, additionally characterized by hexadecanoic acid (4.5%) and (*E*)-geranyl acetone (4.1%). Another common feature of the analyzed oils was the presence of terpenoids (46.8 and 49.5% in *A. uva-ursi* and *V. vitis-idaea* oils, respectively) and fatty acid derived compounds (34.1% - *V. vitis-idaea*, 10.7% - *A. uva-ursi* oil) in high relative amounts. Fatty acids and fatty acid esters (F, 11.8%), and carotenoid derived compounds (CD, 14.1%) represented a significant portion of *A. uva-ursi* oil. The mentioned constituents belonging to F and CD classes were identified in the *V. vitis-idaea* oil as well, but were present in a considerably smaller relative amount.

**Table 1:** Chemical composition of the essential oils extracted from the leaves of *Arctostaphylos uva-ursi* and *Vaccinium vitis-idaea*.

RI^1^	Class	Identification^2^	Compound	*V. vitis-idaea,* %	*A. uva-ursi,* %
725	GL	a, b	(*Z*)-3-Penten-1-ol		tr
732	GL	a, b	(*E*)-3-Penten-2-one	tr^3^	tr
739	MRP	a, b, c	Pyridine		tr
744	GL	a, b	(*E*)-2-Pentenal	tr	tr
762	GL	a, b, c	1-Pentanol	tr	tr
765	GL	a, b	(*Z*)-2-Penten-1-ol	0.8	0.2
772	TH	a, b	3-Methyl-2-buten-1-ol (syn.^4^ prenol)		0.1
772	O	a, b, c	*N,N*-Dimethyl formamide		0.4
781	TH	a, b	3-Methyl-2-butenal (syn. prenal)		tr
783	GL	a, b, c	2,4-Pentandione (syn. acetyl acetone)	tr	
801	GL	a, b, c	Hexanal	tr	0.1
824	MRP	a, b	Methylpyrazine		tr
827	O	a, b, c	Maleic anhydride		tr
832	TH	a, b, c	2-Methylbutanoic acid	tr	
828	GL/MRP	a, b, c	Furfural	0.2	0.8
839	GL	a, b, c	4-Hydroxy-4-methyl-2-pentanone	tr	tr
844	GL	a, b	(*E*)-3-Hexen-1-ol	tr	
854	GL	a, b	(*E*)-2-Hexenal	tr	0.7
854	GL	a, b	(*E*)-2-Hexen-1-ol	1.2	
858	GL	a, b	(*Z*)-3-Hexen-1-ol	4.4	tr
867	GL	a, b, c	1-Hexanol	tr	tr
863	MRP	a, b, c	3-Methylpyridine		0.2
869	MPR	a, b	α-Angelica lactone		tr
892	AE	a, b	1-Nonene		tr
896	O	a, b	2-Methyl-2-cyclopentenone		tr
900	TH	a, b, c	Isopropyl 3-methylbutanoate		0.1
913	GL	a, b	(*E*,*E*)-2,4-Hexadienal	tr	tr
915	MRP	a, b, c	2-Acetylfuran	tr	0.2
916	MRP	a, b	Ethylpyrazine		tr
920	MRP	a, b	2,3-Dimethylpyrazine		tr
935	GL	a, b	2-Methylpentanoic acid	tr	
956	GL	a, b	(*E*)-2-Heptenal	tr	0.1
959	MRP	a, b	3-Ethylpyridine		0.1
959	GL	a, b	(*Z*)-3-Hepten-1-ol	tr	
965	MRP	a, b, c	Benzaldehyde	0.6	0.2
963	MRP	a, b	5-Methyl-2-furancarboxaldehyde		tr
967	GL	a, b, c	1-Heptanol	tr	tr
968	MRP	a, b	3-Ethenylpyridine		0.4
971	GL	a, b, c	Hexanoic acid	0.6	
973	GL	a, b	(*E*)-4-Octen-3-one		tr
978	GL	a, b	1-Octen-3-ol	tr	0.1
978	MRP	a, b, c	Phenol		tr
986	CR	a, b	6-Methyl-5-hepten-2-one		0.1
989	O	a, b, c	Benzonitrile		tr
993	GL	a, b	2-Pentylfuran		tr
995	GL	a, b	3-Octanol		tr
999	GL	a, b	(*E*,*Z*)-2,4-Heptadienal	tr	0.3
1001	MRP	a, b	2-Ethyl-6-methylpyrazine		tr
1004	MRP	a, b	2-Ethyl-5-methylpyrazine		tr
1005	MRP	a, b	Trimethylpyrazine		tr
1013	GL	a, b	(*E*,*E*)-2,4-Heptadienal	tr	0.7
1019	MRP	a, b	5-Ethyl-2-methylpyridine		tr
1022	TMM	a, b, c	Limonene	tr	
1027	O	a, b	2-Ethylhexan-1-ol	tr	0.1
1027	TMA	a, b, c	p-Cymene		tr
1031	GL	a, b	(*E*)-3-Octen-2-one	tr	tr
1036	MRP	a, b, c	Benzyl alcohol	0.7	0.2
1047	MRP	a, b, c	Phenylacetaldehyde	0.5	1.0
1047	O	a, b, c	Salicylaldehyde		tr
1053	O	a, b, c	2-Methylphenol		0.1
1057	A	a, b	4-Methyldecane	tr	
1058	GL	a, b	(*Z*)-2-Octenal		0.1
1062	GL	a, b, c	Pentyl isobutanoate	tr	
1067	GL	a, b	(*E*)-2-Octen-3-ol	tr	0.1
1069	GL	a, b, c	1-Octanol	0.9	0.7
1071	GL	a, b	(*E*,*E*)-3,5-Octadien-2-one		tr
1071	O	a, b, c	Acetophenone	tr	
1072	O	a, b, c	4-Methylbenzaldehyde		tr
1074	O	a, b, c	4-Methylphenol		tr
1075	TMA	a, b, c	*cis*-Linalooloxide (furanoid)	0.5	1.3
1086	O	a, b	3-Methylbenzaldehyde		tr
1090	TMM	a, b	α-Cumyl alcohol (syn. 2-phenyl-2-propanol)	tr	
1092	TMA	a, b, c	*trans*-Linalooloxide (furanoid)	0.3	0.9
1093	TMM	a, b	p-Cymenene		tr
1097	GL	a, b	1-Nonen-4-ol		tr
1098	GL	a, b	Isobutyl tiglate	0.4	
1102	TMA	a, b, c	Linalool	4.7	7.3
1106	GL	a, b, c	Nonanal	0.5	tr
1107	TM	a, b	Hotrienol	tr	
1107	CR	a, b	6-Methyl-3,5-heptadien-2-one		1.8
1111	TMT	a, b, c	α-Thujone		0.7
1114	O	a, b	2,6-Dimethylcyclohexanol	tr	0.2
1117	MRP	a, b, c	2-Phenyl-1-ethanol		0.6
1120	TMA	a, b	Myrcenol		tr
1121	TMT	a, b, c	β-Thujone		0.1
1125	TMT	a, b	Dehydrosabinaketone		0.1
1126	CR	a, b, c	Isophorone	tr	
1130	TMP	a, b	α-Campholenal		tr
1140	GL	a, b	(*E*)-3-Nonen-2-one		tr
1143	O	a, b	Phenylacetonitrile	tr	
1145	TMP	a, b, c	*trans*-Pinocarveol		tr
1145	TMM	a, b	Lilac aldehyde B	tr	0.2
1147	CR	a, b	4-Oxoisophorone	tr	tr
1150	TMB	a, b, c	Camphor		0.8
1154	TMA	a, b	Lilac aldehyde A	0.4	tr
1154	GL	a, b	(*E*,*Z*)-2,6-Nonadienal		0.4
1157	TMA	a, b	Neroloxide		tr
1158	TMM	a, b, c	Menthone		0.2
1161	GL	a, b	(*E*)-2-Nonenal	tr	0.3
1165	TMA	a, b	(Z)-β-Ocimenol		0.1
1167	O	a, b, c	Benzyl acetate	tr	
1169	TMM	a, b, c	Menthol		0.3
1169	TMA	a, b	Lilac aldehyde C	tr	
1170	F	a, b, c	Octanoic acid	0.7	
1171	TMM	a, b	α-Phellandren-8-ol		tr
1172	TMM	a, b	p-Mentha-1,5-dien-8-ol	tr	
1172	TMB	a, b, c	Borneol		1.4
1173	TMA	a, b	*cis*-Linalooloxide (pyranoid)		tr
1175	O	a, b, c	Ethyl benzoate	tr	
1177	TMM	a, b	Isomenthol		1.9
1179	TMA	a, b	*trans*-Linalool oxide (pyranoid)	tr	
1181	O	a, b	2,4-Dimethylbenzaldehyde	tr	
1182	TMM	a, b	Terpinen-4-ol	0.5	1.0
1186	TMP	a, b	Isoverbanol	tr	
1188	TMM	a, b	*neo-*Isomenthol		tr
1189	TMM	a, b	p-Cymen-8-ol	0.2	0.6
1190	O	a, b, c	Naphthalene		tr
1196	TMM	a, b	α-Terpineol	17.0	7.8
1200	O	a, b, c	Methyl salicylate	0.5	0.1
1202	TMP	a, b, c	Myrtenol		0.1
1203	TMM	a, b	γ-Terpineol	tr	
1205	CR	a, b	Safranal	tr	0.2
1207	GL	a, b, c	Decanal	tr	0.1
1213	TMM	a, b	*trans*-Piperitol		tr
1216	TMP	a, b, c	Verbenone		0.3
1216	GL	a, b	(*E*,*E*)-2,4-Nonadienal	tr	tr
1221	TMM	a, b	1-p-Menthen-9-al isomer 1	0.4	0.6
1223	TMM	a, b, c	*trans*-Carveol		tr
1223	TMM	a, b	1-p-Menthen-9-al isomer 2	0.5	0.4
1226	CR	a, b	β-Cyclocitral	0.4	0.2
1231	TMA	a, b	(*Z*)-Ocimenone	tr	
1231	TMA	a, b, c	Nerol	0.2	0.8
1237	TMM	a, b, c	Thymol methyl ether		
1244	TMM	a, b, c	Pulegone		0.3
1247	TMM	a, b, c	Carvacrol methyl ether	tr	
1249	TMM	a, b, c	Carvone		0.2
1252	TMM	a, b	Perilla ketone		0.1
1256	TMA	a, b, c	Geraniol	1.5	3.0
1259	TMM	a, b	Piperitone		0.2
1263	GL	a, b	(*E*)-2-Decenal	0.5	0.7
1273	TMA	a, b, c	Geranial		1.3
1275	F	a, b, c	Nonanoic acid	0.4	0.7
1277	TMM	a, b	Perilla aldehyde	1.2	
1286	GL	a, b	Vitispirane		0.9
1290	PP	a, b, c	*trans*-Anethole		0.6
1290	TMB	a, b, c	Isobornyl acetate		tr
1294	TMM	a, b, c	Thymol		2.0
1294	AE	a, b	1-Tridecene	tr	
1296	GL	a, b	(*E*,*Z*)-2,4-Decadienal	tr	tr
1297	TMM	a, b, c	Menthyl acetate		tr
1299	O	a, b	2-Methylnaphthalene		tr
1300	O	a, b, c	Indole	tr	
1300	A	a, b, c	Tridecane	tr	
1304	TMM	a, b, c	Carvacrol		0.9
1304	TMM	a, b	Perilla alcohol	tr	
1309	GL	a, b	Undecanal		0.1
1313	CR	a, b	Riesling acetal		1.4
1317	O	a, b	1-Methylnaphthalene		tr
1318	PP	a, b	4-Vinylguaiacol	tr	tr
1319	GL	a, b	(*E*,*E*)-2,4-Decadienal	0.8	1.7
1323	O	a, b	2,4,6-Trimethylbenzaldehyde		0.1
1326	GL	a, b	(*Z*)-3-Hexenyl tiglate	tr	
1336	A	a, b	Branched alkane	tr	
1341	CR	a, b	(*E*,*E*)-2,5-Epoxy-6,8-megastigmadiene	tr	
1344	A	a, b	Branched alkane	tr	
1353	TMM	a, b	α-Terpineol acetate		0.7
1359	O	a, b	1,1,6-Trimethyl-1,2-dihydronaphthalene	0.2	0.6
1361	PP	a, b, c	Eugenol	0.7	tr
1363	TMA	a, b	Hydroxy citronellol(syn. 3,7-dimethyl-1,7-octanediol)	tr	
1366	GL	a, b	(E)-2-Undecenal	tr	0.2
1367	F	a, b	γ-Nonalactone	tr	
1371	CR	a, b	(*E*,*Z*)-4,6,8-Megastigmatriene	tr	
1370	F	a, b, c	Decanoic acid	tr	0.8
1377	A	a, b	3-Methyltridecane	tr	
1383	CR	a, b	α-Ionol		0.3
1383	GL	a, b	(*Z*)-3-Hexenyl hexanoate	0.8	
1384	O	a, b, c	Biphenyl		tr
1388	GL	a, b	(*Z*)-3-Hexenyl (*Z*)-3-hexenoate	0.3	
1390	CR	a, b	(*E*)-β-Damascenone		0.3
1391	GL	a, b	(*E*)-2-Hexenyl caproate	tr	
1396	AE	a, b	1-Dodecene	tr	
1398	CR	a, b	(*Z*)-Jasmone	tr	
1400	A	a, b, c	Tetradecane	tr	tr
1404	CR	a, b	(2*E*)-3-(2,6,6-trimethyl-1-cyclohexen-1-yl)-2-propenal	tr	0.4
1407	CR	a, b	Hexahydropseudoionone (syn. tetrahydrogeranyl acetone)	tr	0.1
1409	O	a, b	2,6-Dimethylnaphthalene		tr
1411	AL	a, b	Dodecanal	tr	tr
1416	O	a, b	1-Ethenylnaphthalene		tr
1420	CR	a, b	(*E*)-β-Damascone	tr	
1423	TS	a, b	β-Cedrene		0.2
1424	O	a, b	1,3-Dimethylnaphthalene		tr
1427	TSCR	a, b, c	β-Caryophyllene	2.9	0.9
1433	CR	a, b, c	(*E*)-α-Ionone		0.1
1440	TS	a, b	Calarene (syn. β-Gurjunene)		tr
1444	O	a, b	2,3-Dimethylnaphthalene		tr
1454	O	a, b	Acenaphthylene		tr
1456	CR	a, b	(*E*)-Geranyl acetone	tr	4.1
1457	A	a, b	4-Methylpentadecane	tr	
1460	TSF	a, b	(*E*)-β-Farnesene		tr
1462	TSH	a, b, c	α-Humulene	1.1	1.2
1463	A	a, b	2-Methyltetradecane	tr	tr
1465	F	a, b	Undecanoic acid		0.2
1476	ALC	a, b	1-Dodecanol		tr
1483	TSCD	a, b	γ-Muurolene		0.6
1488	TSGER	a, b	Germacrene D		tr
1492	CR	a, b, c	(*E*)-β-Ionone	1.1	1.3
1494	TSED	a, b	β-Selinene		tr
1497	TS	a, b	α-Zingiberene	0.6	
1498	FAD	a, b	2-Tridecanone		0.1
1500	TSED	a, b	δ-Selinene	tr	
1500	A	a, b, c	Pentadecane	tr	tr
1503	TSED	a, b	α-Selinene		0.8
1503	O	a, b	Benzyl tiglate	tr	
1507	TSCD	a, b	α-Muurolene		0.2
1509	TSAG	a, b	4-*epi-cis*-Dihydroagarofuran	tr	
1511	TSF	a, b	(*E*,*E*)-α-Farnesene	4.9	
1513	AL	a, b	Tridecanal		tr
1513	TS	a, b	β-Bisabolene		0.3
1514	TSCD	a, b	γ-Cadinene		tr
1521	TSCD	a, b	δ-Cadinene		0.9
1526	TSED	a, b	7-*epi*-α-Selinene	tr	
1527	PP	a, b, c	Myristicin		0.6
1530	TSCD	a, b	*trans*-Cadina-1,4-diene		tr
1532	O	a, b	Lilial	tr	
1535	CR	a, b	(*E*,*Z*)-Pseudoionone		0.3
1538	CR	a, b	Dihydroactinidiolide		0.1
1542	A	a, b	Branched alkane	tr	
1544	TSCD	a, b	α-Cadinene		tr
1550	TSCD	a, b	α-Calacorene		0.2
1555	TSAG	a, b	α-Agarofuran	1.8	
1561	A	a, b	2-Methylpentadecane	tr	
1565	F	a, b, c	Dodecanoic acid	0.6	1.8
1571	O	a, b	2,3,5-Trimethylnaphthalene		0.2
1571	A	a, b	3-Methylpentadecane		tr
1576	GL	a, b	(*Z*)-3-Hexenyl benzoate	tr	0.3
1582	TSF	a, b	(*Z*)-Dihydroapofarnesol	0.8	
1586	TS	a, b, c	Spathulenol		tr
1587	O	a, b	9*H*-Fluorene		0.6
1589	CR	a, b	(*E*,*E*)-Pseudoionone		0.4
1592	TSCR	a, b, c	Caryophyllene oxide	0.8	
1593	AE	a, b	1-Hexadecene	tr	
1594	O	a, b	3,3'-Dimethylbiphenyl		tr
1600	A	a, b, c	Hexadecane		0.7
1601	TS	a, b	Viridiflorol		0.1
1602	TS	a, b	4(14)-Salvialen-1-one		tr
1607	TSH	a, b	Humulene epoxide I		0.1
1611	TS	a, b	Cedrol		0.2
1614	AL	a, b	Tetradecanal	tr	
1615	AL	a, b	(*E*)-7-Tetradecenal		tr
1619	TSH	a, b	Humulene epoxide II		tr
1629	F	a, b, c	Isopropyl laurate		tr
1629	TSED	a, b	10-*epi*-γ-Eudesmol	3.0	
1634	O	a, b, c	Benzophenone		tr
1638	O	a, b	4-Methyldibenzofuran		tr
1640	TSED	a, b	γ-Eudesmol	tr	0.6
1646	TSCR	a, b	Caryophylla-3(15),7(14)-dien-6-ol		tr
1649	TSCD	a, b	τ-Cadinol		0.3
1650	TSCD	a, b	Cubenol		tr
1654	TSCD	a, b	α-Muurolol		0.2
1659	TSED	a, b	α-Eudesmol	tr	
1662	TSER	a, b	Valerianol	1.7	
1665	A	a, b	2-Methylhexadecane		tr
1666	TSCD	a, b	α-Cadinol		0.2
1667	TSED	a, b	7-*epi*-α-Eudesmol	tr	
1675	ALC	a, b	1-Tetradecanol	0.9	
1676	TSF	a, b	Hexahydrofarnesol		0.1
1683	O	a, b	Hexyl salicylate	tr	
1694	AE	a, b	1-Heptadecene	tr	tr
1695	TSCD	a, b	Amorpha-4,9-dien-2-ol		0.1
1698	TS	a, b	Acorenone		0.2
1700	A	a, b, c	Heptadecane	0.3	0.2
1705	TSGER	a, b, c	Germacrone	1.1	
1716	AL	a, b	Pentadecanal	0.3	0.1
1719	TSF	a, b	(*E*,*E*)-Farnesal		tr
1725	O	a, b	2,6-Diisopropylnaphthalene		0.2
1727	F	a, b, c	Methyl tetradecanoate	tr	
1755	A	a, b	5-Methylheptadecane	tr	
1764	A	a, b	2-Methylheptadecane	tr	
1765	F	a, b, c	Tetradecanoic acid		1.2
1772	O	a, b, c	Benzyl benzoate	tr	0.2
1784	O	a, b, c	Phenanthrene		0.1
1794	AE	a, b	1-Octadecene	tr	0.1
1795	F	a, b, c	Ethyl tetradecanoate	tr	
1800	A	a, b, c	Octadecane		0.1
1818	AL	a, b	Hexadecanal	tr	tr
1828	F	a, b, c	Isopropyl myristate	tr	0.1
1839	F	a, b	15-Pentadecanolide (syn. exaltolide)	tr	
1844	TSF	a, b	(*E*,*E*)-2,6-Farnesyl acetate	tr	
1848	CR	a, b	Hexahydrofarnesyl acetone	1.7	2.3
1862	F	a, b	Pentadecanoic acid		tr
1876	O	a, b, c	Benzyl salicylate	tr	tr
1883	ALC	a, b, c	1-Hexadecanol		tr
1894	AE	a, b	1-Nonadecene	tr	
1900	A	a, b, c	Nonadecane	tr	0.1
1921	CR	a, b	(*E*,*E*)-5,9-Farnesyl acetone	tr	0.7
1928	F	a, b, c	Methyl hexadecanoate	tr	tr
1930	O	a, b	2-Methylanthracene		tr
1941	F	a, b	(*Z*)-9-Hexadecenoic acid (syn. palmitoleic acid)		0.2
1950	TD	a, b	Isophytol	tr	0.2
1968	F	a, b, c	Hexadecanoic acid	tr	4.5
1975	TD	a, b	Sandaracopimara-8(14),15-diene	tr	
1982	ALC	a, b, c	1-Heptadecanol	tr	
1994	AE	a, b	1-Eicosene	tr	tr
1996	F	a, b, c	Ethyl hexadecanoate	tr	0.2
2000	A	a, b, c	Eicosane	tr	0.1
2003	TD	a, b	Manoyl oxide	1.4	0.1
2025	TD	a, b	13-*epi*-Manool oxide	2.0	
2034	CR	a, b	(*E*,*E*)-Geranyl linalool	tr	
2070	TD	a, b	ar-Abietatriene	tr	
2094	AE	a, b	1-Heneicosene	tr	tr
2100	A	a, b, c	Heneicosane	0.3	0.1
2116	TD	a, b	(*E*)-Phytol	tr	3.3
2116	AL	a, b	Nonadecanal	tr	
2136	AE	a, b, c	Linoleic acid		0.3
2138	A	a, b	Branched alkane	tr	
2143	F	a, b, c	Linolenic acid		1.2
2155	F	a, b	(*E*,*E*)-9,12-Octadecadienoic acid (syn. linoleic acid)		0.8
2172	AE	a, b	1-Nonadecanol	tr	
2180	A	a, b	Branched alkane	tr	
2194	AE	a, b	1-Docosene	0.8	tr
2200	A	a, b, c	Docosane	0.5	tr
2219	AL	a, b	Eicosanal		tr
2294	AE	a, b	1-Tricosene	0.3	
2300	A	a, b, c	Tricosane	2.1	0.2
2395	AE	a, b	1-Tetracosene	1.4	
2352	F	a, b	δ-Octadecalactone	tr	0.1
2394	AE	a, b	1-Tetracosene		tr
2400	A	a, b, c	Tetracosane	1.2	tr
2495	AE	a, b	1-Pentacosene	tr	
2500	A	a, b, c	Pentacosane	6.4	tr
2596	AE	a, b	1-Hexacosene	1.1	
2600	A	a, b, c	Hexacosane	0.6	
2700	A	a, b, c	Heptacosane	2.9	
2297	AE	a, b	1-Octacosene	1.6	
2900	A	a, b, c	Nonacosane	2.0	
2998	AE	a, b	1-Triacontene	tr	
3100	A	a, b, c	Hentriacontane	tr	
			**Total**	**91.7**	**90.4**
			**Number of constituents**	**187**	**243**
			**Terpenoids (T)**	**49.5**	**46.8**
			**Hemiterpenoids (TH)**	**tr**	**0.2**
			**Monoterpenoids (TM)**	**27.4**	**35.6**
			Oxygenated	27.4	35.6
			Hydrocarbons	tr	tr
			Acyclic (TMA)	7.6	14.7
			p-Menthane (TMM)	19.8	17.4
			Bornane (TMB)	0.0	2.2
			Thujane (TMT)	0.0	0.9
			Pinane (TMP)	tr	0.4
			**Sesquiterpenoids (TS)**	**18.7**	**7.4**
			Oxygenated	9.2	2.1
			Hydrocarbons	9.5	5.3
			Farnesane (TSF)	5.7	0.1
			Caryophyllane (TSCR)	3.7	0.9
			Eudesmane (TSED)	3.0	1.4
			Cadinane (TSCD)	0.0	2.7
			Germacrane (TSGER)	1.1	tr
			Eremophylane (TSER)	1.7	0.0
			Salvialane, acorane, bisabolane, aromadendrane, cedrane, gurjunane (TS)	0.6	1.0
			Agarofurane (TSAG)	1.8	0.0
			Humulane (TSH)	1.1	1.3
			**Diterpenoids (TD)**	**3.4**	**3.6**
			**Phenylpropanoids (PP)**	**0.7**	**1.2**
			**Fatty acid derived compounds (FAD)**	**34.1**	**10.7**
			**Alkanes (A)**	**16.3**	**1.5**
			**Alkenes (AE)**	**5.2**	**0.4**
			**Aldehydes (AL)**	**0.3**	**0.1**
			**Alcohols (ALC)**	**0.9**	**tr**
			**“Green leaf” volatiles (GL)**	**11.4**	**8.6**
			**Fatty acids and fatty acid esters (F)**	**1.7**	**11.8**
			**Carotenoid derived compounds (CD)**	**3.2**	**14.1**
			**Maillard reaction products (MRP)**	**1.8**	**2.9**
			**Others**	**0.7**	**2.9**

^1 ^Compounds listed in order of elution on HP-5MS column (RI- experimentally determined retention indices on the mentioned column by co-injection of a homologous series of *n*-alkanes C_7_-C_29_); ^2^ a: constituent identified by mass spectra comparison; b: constituent identified by retention index matching; c: constituent identity confirmed by co-injection of an authentic sample; ^3 ^tr- trace (<0.05%); ^4^ syn.-synonym.

In respect to the skeleton-types of the identified constituents, the monoterpenoid fractions of both *V. vitis-idaea* and *A. uva-ursi* oils could be considered as rather simple. Interestingly, not taking into account some trace constituents, the monoterpenoid fractions of both oils were completely comprised of oxygenated derivatives. In *V. vitis-idaea* oil only acyclic (7.6%), *p*-menthane (19.8%) and pinane-type (tr) monoterpenoids were detected. Acyclic (14.7%) and monoterpenoids with a *p*-menthane (17.4%) skeleton dominated the monoterpenoid fraction of *A. uva-ursi* oil as well, and only small relative amounts of pinane (0.4%), bornane (2.2%) and thujane-type (0.9%) compounds were detected. α-Terpinyl cation, produced by the biosynthetic cyclization of linalyl diphosphate, the intermediate from which p-menthane type monoterpenoids are derived, is known to be the precursor of other classes of cyclic monoterpenoids including bornanes, pinanes and thujanes [24]. Biosynthesis of linalool is closely related to linalyl diphosphate, and α-terpineol could be considered as a direct biosynthetic product of α-terpinyl cation, formed by quenching the mentioned cation with water [24]. Both linalool and α-terpineol were by far the most abundant compounds in the monoterpenoid fractions of *A. uva-ursi* and *V. vitis-idaea* oils. Having the above mentioned in mind, one could speculate that both taxa have a relatively primitive monoterpenoid biosynthetic “apparatus”, capable of producing predominantly metabolites from the “beginning” of the mentioned metabolic pathway. It seems that a similar consideration stands for some other taxa from the genus *Vaccinium* as well. In different *Vaccinium* species (*V. corrymbosum*, *V. oxycoccus*, *V. macrocarpon*, *V. arctostaphylos*) α-terpineol and/or linalool were recognized as major, or one of the major volatile metabolites [14,25,26,27,28,29]. Nevertheless, this should be taken with a grain of salt, since only the volatile metabolites have been investigated. In the studied species monoterpenes could be potentially present as glycosides, and thus non-volatile under hydrodistillation and/or GC conditions. α-Terpineol and some other terpenoid compounds were previously also recognized as *V. macrocarpon* cuticle wax constituents [30]. According to Croteau *et al*., the presence of these compounds in the cuticle wax could suggest that these substances might have a certain role in the plants’ defense mechanisms [30]. 

Although the relative amount of volatile sesquiterpenoids was considerably lower than that of monoterpenoids in both *A. uva-ursi* and *V. vitis-idaea* oils, sesquiterpenoid fractions were, concerning skeleton-types of identified constituents, much more heterogenic (Table 1). A number of different skeleton-types of volatile sesquiterpenoids were dominant in the oils of the two taxa: farnesanes (5.7%), caryophyllanes (3.7%) eudesmanes (3.0%) in *V. vitis-idaea* oil and cadinanes (2.7%), eudesmanes (1.4%) and humulanes (1.3%) in *A. uva-ursi* oil.

It might be assumed that certain volatiles listed in Table 1, identified in both *A. uva-ursi* and *V. vitis-idaea* oils, could be considered artifacts of the isolation procedure, and not direct products of plant metabolism. For example, a number of compounds from the Table 1 are most probably products of Maillard-type reactions including the thermal fragmentation of amino acids and sugars, alone or in conjunction, during hydrodistillation [31]. “Green leaf” volatiles, on the other hand, are most probably produced by enzymatic degradation of unsaturated fatty acids, as in desiccation, *i.e.* as a stress-induced response of plants, produced during collection and preparation of plant samples [32]. Alongside “green leaf” and other fatty acid derived compounds (FAD), fatty acid and fatty acid esters (F) and carotenoid derived compounds (CD) represented more than one third of both analyzed oils. Volatile profiles of some other representatives of the genus *Vaccinium* were also dominated by FAD, F and/or CD compounds [13,33,34]. All these species could be considered as essential oil-poor species (oil yield less than 0.1%). All mentioned above seems to further corroborate the hypothesis proposed by us in a previous publication [10]. We have noticed that the correlation between the essential oil yield and composition (classes of compounds) exists [10]. Most frequently, essential oil-rich species (oil yield much higher than 0.1%) produce considerable amount of monoterpenoids or phenylpropanoids, while in the oils of essential oil-poor species, FAD, F and CD compounds are the dominant volatile metabolites [10]. 

As previously mentioned, there are no reports concerning the volatile metabolites of *A. uva-ursi*, and there are only two references on *V. vitis-idaea* volatiles, however different parts of the plant (berries instead of leaves), using a different methodology (minced berries were treated with a pectinolytic enzyme and after that volatiles of the obtained juice and pressed residue were separately studied), have been analyzed [11,12]. Volatile profile of *V. vitis-idaea* barriers differs significantly from the corresponding profile of the leaves. For example, the most dominant volatile of the pressed residue of minced berries was benzyl alcohol (40.2%), found only as the minor contributor of the leaves’ oil. α-Terpineol and linalool (dominant volatiles of *V. vitis-idaea* leaves) on the other hand, represented in total only 1.0% of berry extract. This plant organ specification, concerning production/accumulation of volatiles, is not unusual. For example, differences in the chemical composition of *Artemisia absinthium* root and aerial parts oils pointed out to the possibility that different metabolic pathways could be operational in different organs of the same plant species [35]. Still, some similarities between *V. vitis-idaea* berry and leaf essential oil profiles could be observed. For instance, fatty acid related compounds, one of the dominant groups of constituents in the leaf oil, represented a significant portion of the berry extract (*ca.* 20%) [11,12].

Both species analyzed herein belong to the plant family Ericaceae. The latter comprises some 100-125 genera and more than 3,000 species [36] that are, generally speaking, poorly studied in respect to volatile metabolites. Table 2 lists the Ericaceae taxa whose essential oils were previously chemically analyzed using a methodology comparable to that applied in this work [13,14,15,16,17,18,19,20,21,22,23]. Compositional data on the essential oils of the species listed in Table 2 (28 samples in total) were mutually compared by means of multivariate statistical analyses (MVA: AHC and PCA).

**Table 2:** List of essential oil samples used in statistical analyses.

Taxon (plant part)	Main oil constituent	Ref.^1^	Des.^2^
*Arctostaphylos uva-ursi* (L.) Spreng. (leaves)	α-Terpineol (7.8%)	Present study	Obs1
*Vaccinium vitis-idaea* (leaves)	α-Terpineol (17.0%)	Present study	Obs2
*V. arctostaphylos* L. (shoots)	Hexadecanoic acid (27.0%)	[13]	Obs3
*V. arctostaphylos* (aerial parts)	α-Terpineol (15.0%)	[14]	Obs4
*Rhododendron mucronatum* G. don (flowers)	Linolenic acid (39.7%)	[15]	Obs5
*R. simii* Planch. (flowers)	Linolenic acid (36.4%)	[15]	Obs6
*R. simii* (leaves)	Phytol^3^ (15.2%)	[15]	Obs7
*R. naamkwanense* Merr. (leaves)	9,12-Octadecatienoic acid (45.3%)^4^	[15]	Obs8
*R. anthopogon* D. Don (aerial parts)	α-Pinene (37.4%)	[16]	Obs9
*R. aureum* Georgi. (leaves)	Calarene (34.4%)	[17]	Obs10
*R. aureum* (leaves)	Calarene (66.4%)	[17]	Obs11
*R. aureum* (leaves)	Calarene (26.2%)	[17]	Obs12
*R. aureum* (leaves)	Calarene (41.3%)	[17]	Obs13
*R. aureum* (leaves)	β-Bourbonene (27.4%)	[17]	Obs14
*R. aureum* (leaves)	Calarene (48.8%)	[18]	Obs15
*R. aureum* (leaves)	Calarene (36.2%)	[18]	Obs16
*R. aureum* (leaves)	Calarene (16.2%)	[18]	Obs17
*R. dauricum* L. (leaves)	*trans*-Caryophyllene (19.1%)	[18]	Obs18
*R. dauricum* (leaves)	γ-Cadinene (17.4%)	[18]	Obs19
*R. dauricum* (leaves)	*trans*-Caryophyllene (17.0%)	[18]	Obs20
*R. tomentosum* (Stokes) H. Harmaja (leaves) (former name *Ledum palustre* L.)	Palustrol (22.8%)	[19]	Obs21
*Ledum palustre* L. var. *angustum* N. Busch	Ascaridole (26.8%)	[20]	Obs22
*Erica manipuliflora* Salisb. (aerial parts)	Heptacosane (19.9%)	[21]	Obs23
*E. manipuliflora* (aerial parts)	1-Octen-3-ol (16.2%)	[21]	Obs24
*Gaultheria fragrantissima* Wall. (leaves)	Methyl salicylate (99.2%)	[22]	Obs25
*G. fragrantissima* (steams)	Methyl salicylate (99.5%)	[22]	Obs26
*G. fragrantissima* (flowering twigs)	Methyl salicylate (99.4%)	[22]	Obs27
*Arbutus unedo* L. (leaves)	(*E*)-2-Decenal (12.0%)	[23]	Obs28

^1^ Ref.-reference; ^2 ^Des.-designation;^ 3 ^Correct isomer not specified in the original reference; ^4 ^Name of component (incomplete and unclear) given as in the original reference.

**Figure 1:**
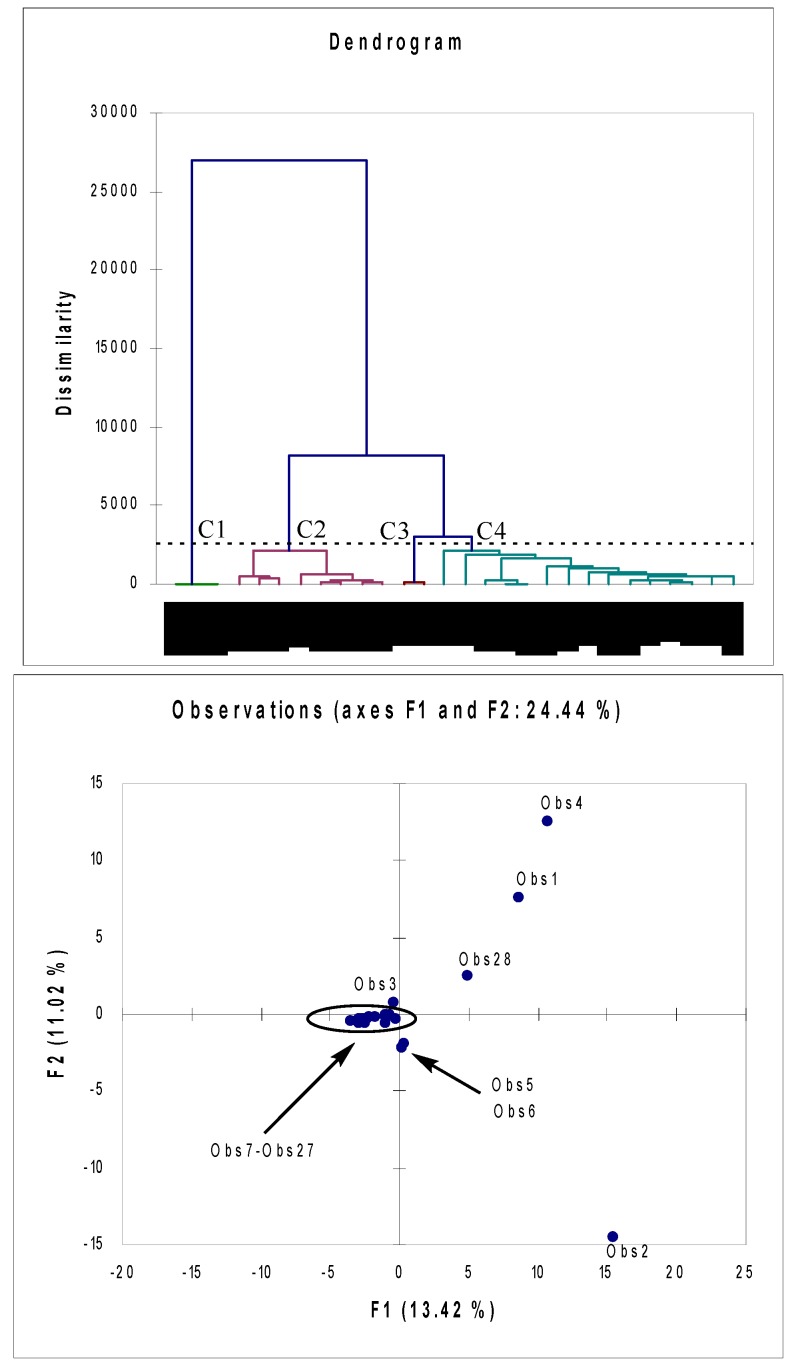
**(a)** Dendrogram (AHC analysis) representing chemical composition dissimilarity relationships of 28 essential oil samples (observations) obtained by Euclidian distance dissimilarity (dissimilarity within the interval [0, 27000], using aggregation criterion-Ward's method). Four groups of samples (C1-C4) were found. **(b)** Principal component analysis ordination of 28 oil samples (observations). Axes (F1 and F2 factors-the first and second principal component) refer to the ordination scores obtained from the samples. Axis F1 accounts for *ca.* 13% and axis F2 accounts for a further 11% of the total variance.

This was done in order to determine, based on the essential oil profiles, possible mutual alliance of the taxa within the family, especially that of species from the genera *Arctostaphylos* and *Vaccinium*. Principal component analysis (PCA) and agglomerative hierarchical clustering (AHC) were both performed using the Excel program plug-in XLSTAT version 2008.6.07. Both methods were applied utilizing the mean values of the relative abundances of the constituents of compared essential oils as variables (only constituents with percentage higher than 1% in at least one sample were taken into account). AHC was determined using Pearson dissimilarity where the aggregation criterion were simple linkage, unweighted pair-group average and complete linkage and Euclidean distance where the aggregation criterion were weighted pair-group average, unweighted pair-group average and Ward’s method. PCA of the Pearson (n) type was performed. Results of the MVA analyses are given in Figure 1 and Figure 2. In the dendrogram of the AHC analysis (Figure 1), four different classes of samples (C1-C4) can be observed. Class C1 (Obs25-Obs27) groups essential oils (almost pure methyl salicylate) obtained from different parts of *Gaultheria fragrantissima* (wintergreen) [22]. Class C2 consists exclusively of *Rhododendron aureum* oils (Obs10-Obs17), all characterized with a high level of the sesquiterpene calarene [17,18]. Oils obtained from flowers of *R. mucronatum* and *R. simii* (Obs5 and Obs 6; high level of linolenic acid) form a separate class C3 [15]. All other samples [13,14,15,16,17,18,19,20,21,22,23], including those that correspond to *A. uva-ursi* (Obs1), *V. vitis-idaea* (Obs2) and *V. arctostaphylos* (Obs3 and Obs4) are recognized as statistically not different and grouped in C4. It must be stressed that samples Obs1-Obs4 are basically characterized by very low Euclidian distance. In the same time, different species from the genus *Rhododendron* are separated in statistically different groups (C2, C3 and C4) [15,20]. Results of AHC suggest that taxa from the genera *Vaccinium* and *Arctostaphylos* are closely related. This is observable from the PCA biplot as well (Figure 2), where *A. uva-ursi* (Obs1) and *V. arctostaphylos* (Obs4) oils are mutually characterized with similar values of F1 and F2 factors. Moreover, samples corresponding to *Vaccinium, Arctostaphylos* and *Arbutus* taxa (Obs1, Obs2, Obs4 and Obs28) are, based on PCA results, clearly separated from other considered oils (Figure 2). One could find results of both AHC and PCA a bit surprising, having in mind that classical taxonomy places genera *Vaccinium* and *Arctostaphylos* in different subfamilies of Ericaceae (Vaccinoidaea and Arbutoidaea) [37]. Results of molecular studies within the Ericaceae clearly separated taxa belonging to the mentioned genera [37,38]. Nevertheless, mutual alliance of *Arbutus* and *Arctostaphylos* (Arbutoidaea; Obs1, Obs22) is recognized by both molecular [37,38] and chemotaxonomical studies (present work).

## 3. Experimental

### 3.1. Plant material

Leaves of *V. vitis-idaea* were collected from the slopes of Stara Planina Mountain (near the mountain top Babin Zub), S. Serbia, at the beginning of July, 2007. Voucher specimens were deposited in the Herbarium of the Faculty of Science and Mathematics, University of Niš, under acquisition number 20074. Leaves of *A. uva-ursi* were obtained from a local pharmacy (in 2006). Botanical identification was performed by N.R.

### 3.2. Isolation of the essential oils

Air-dried, to constant weight, leaves of *A. uva-ursi* and *V. vitis-idaea* (three batches of about 500 g of each sample) was subjected to hydrodistillation with *ca*. 2 L of distilled water for 2.5 h using the original Clevenger-type apparatus [39]. The semi-solid yellowish essential oils (30 ± 1 mg per batch) of both species were obtained with a yield of 0.06% (w/w, typical value). Due to the small sample size of 30 mg of the isolated essential oils, which were not completely liquid, the volume of the oils was not measured. The obtained oils were separated by extraction with diethyl ether (Merck, Darmstadt Germany) and dried over anhydrous sodium sulfate (Aldrich, St. Louis, MO, USA). The solvent was evaporated under a gentle stream of nitrogen at room temperature, in order to exclude any loss of the essential oil, and immediately analyzed. When the oil yields were determined, after the bulk of ether was removed under a stream of N_2_, the residue was exposed to *vacuum* at room temperature for a short period to eliminate the solvent completely. The pure oil was then measured on an analytical balance and multiple gravimetric measurements were taken during 24 h to ensure that all of the solvent had evaporated.

### 3.3. Gas chromatography and gas chromatography-mass spectrometry

The GC/MS analysis was repeated three times for each sample using a Hewlett-Packard 6890N gas chromatograph. The gas chromatograph was equipped with a fused silica capillary column HP-5MS (5% phenylmethylsiloxane, 30 m × 0.25 mm, film thickness 0.25 μm, Agilent Technologies, Palo Alto, CA, USA) and coupled with a 5975B mass selective detector from the same company. The injector and interface were operated at 250 ºC and 300 ºC, respectively. The oven temperature was raised from 70 ºC to 290 ºC at a heating rate of 5 ºC/min and then isothermally held for 10 min. As a carrier gas helium at 1.0 mL/min was used. The samples, 1 μL of the oil solutions in diethyl ether (1:100), was injected in a pulsed split mode (the flow was 1.5 mL/min for the first 0.5 min and then set to 1.0 mL/ min throughout the remainder of the analysis; split ratio 40:1). mass selective detector was operated at the ionization energy of 70 eV, in the 35–500 amu range with a scanning speed of 0.34 s. GC (FID) analysis was carried out under the same experimental conditions using the same column as described for the GC/MS. The percentage composition was computed from the GC peak areas without the use of correction factors. Qualitative analysis of the essential oil constituents was based on several factors. Firstly, the comparison of the essential oils linear retention indices relative to retention times of C_7_-C_31_ n-alkanes on the HP-5MS column [40] with those reported in the literature [41]. Secondly, by comparison of their mass spectra with those of authentic standards, as well as those from Wiley 6, NIST02, MassFinder 2.3. Also, a homemade MS library with the spectra corresponding to pure substances and components of known essential oils was used, and finally, wherever possible, by coinjection with an authentic sample (Table 1). Relative standard deviation (RSD) of repeated measurements (independent sample preparations and GC-MS) was for all substances below 1%. The only exceptions which had higher RSD were minor components such as α-agarofuran, (*E*)-β-ionone, pulegone, safranal and dodecanoic acid where RSD was 2, 6, 7, 9 and 12%, respectively.

## 4. Conclusions

Comparison of the compositional data of the essential oils extracted from *A. uva-ursi*, *V. vitis-idaea* and 12 other Ericaceae taxa (six different genera; available literature data) pointed out to a high level of similarity of *Vaccinium* and *Arctostaphylos* species. Based on the identity and relative abundance of the dominant volatile metabolites produced by the compared Ericaceae taxa, it seems that the level of mutual correspondence between *A. uva-ursi* and *V. vitis-idaea* species is more significant than that of any of the two taxa and the rest of the compared species. This is partially due to the fact that α-terpineol and linalool were among the dominant contributors to the volatile profiles of both *A. uva-ursi* and *V. vitis-idaea*. Furthermore, the most abundant classes of compounds in both oils were basically the same (monoterpenoids, fatty acid derived compounds and carotenoid derived compounds). All stated above additionally corroborates the same pharmacological applications of two herbs. It must be stressed once again that essential oils may, despite the small yield, contribute to the medicinal properties of the plant [9].
